# Robotic bimanual arm training improves upper limb function in children with hemiparesis: a feasibility study

**DOI:** 10.3389/fped.2025.1502481

**Published:** 2025-11-19

**Authors:** Talita Campos, Shivakeshavan Ratnadurai-Giridharan, Marion-Victoria Wairimu, Annie Li, Sara Tamayo, Kathleen M. Friel

**Affiliations:** 1 Department of Nursing, Columbia University, New York, NY, United States; 2 Clinical Laboratory for Early Brain Injury, Burke Neurological Institute, White Plains, NY, United States; 3 Brain Mind Research Institute, Weill Cornell Medicine, New York, NY, United States; 4 Mercy University, School of Health & Natural Sciences, Dobbs Ferry, NY, United States

**Keywords:** cerebral palsy, acquired brain injury, hemiplegia, upper extremity, pediatrics, rehabilitation

## Abstract

**Introduction:**

In children with hemiparesis, bimanual training can produce greater gains in upper limb function compared with unimanual training of the impaired upper limb. Moreover, repetitive skilled practice is a critical component of effective interventions. The objective of this study was to determine whether upper limb training using a bimanual-to unimanual robotic training device is feasible and effective for improving hand function in children with hemiplegia. We hypothesized that this robotic training would improve motor outcomes on the Assisting Hand Assessment (AHA), the Box and Blocks Test (BBT), and the Jebsen-Taylor Test of Hand Function (JTTHF).

**Methods:**

Children (*n* = 8, 6 males, 2 females, age 5-17 years) with hemiparesis participated in a feasibility study using a robotic device called the Bimanual Arm Trainer (BAT, Mirrored Motion Works). This device encourages mirrored bimanual movements and uses an engaging gaming interface to encourage repetitive movements. The BAT provides bimanual-to-unimanual training of yoked shoulder external rotation and elbow extension, as well as training of pronation, supination and grasp and release of each hand independently*.*

**Results:**

Although the study was halted by the COVID-19 pandemic, eight children completed 18 training sessions on the BAT (2x/week for 9 weeks). While range of motion did not change significantly, children significantly improved in bimanual (AHA) function.

**Discussion:**

Children enjoyed the device, and provided feedback that was used to improve the gaming environment. Further work is needed to determine ideal dosing to optimize improvements.

## 1 Introduction

Despite decades of neurorehabilitation research, therapies for children with cerebral palsy (CP) remain unable to fully ameliorate the impairments that impact these children's quality of life and ability to fully participate in their communities. The most effective interventions for upper limb weakness involve repetitive, skillful training of therapeutic movements [review (1)].

A challenge in providing an intensive approach to children is sustaining their engagement across the number of repetitions needed to drive large gains in performance. Studies in animal models of stroke (2, 3) and CP (4, 5) demonstrate that intensive skill training, with hundreds of repetitions per day, is necessary to drive movement recovery and adaptive neuroplastic changes to the brain and spinal cord. Unfortunately, most therapies for people with CP do not involve hundreds of repetitions of skilled movements per day. Lang et al. studied the number of movement repetitions completed in therapy sessions for stroke survivors. They found that the average number of repetitions of upper limb movements was 32 per session, far lower than the hundreds of repetitions performed by animals (6). A subsequent study showed that delivering hundreds of repetitions per session in stroke survivors is indeed feasible (7). This encourages the neurorehabilitation community to design and implement high intensity therapies.

Although the number of movement repetitions in a typical therapy session for children with CP has not been studied, the number of repetitions needed to drive robust, long-lasting improvements is far higher than the handful of hours per week most children with CP receive in therapies in the United States. We need to deliver high-intensity, sustained repetition of therapeutic movements to children, in an engaging, cost-effective way that does not put undue stress on participants and their families.

Children with unilateral CP present with hemiparesis, or weakness on one side of the body. One of the most effective interventions for children with hemiparesis is bimanual training [reviews (1, 8, 9)]. The reason why bimanual training is such a valuable approach for children with hemiparesis is because a majority of activities of daily living require good bimanual coordination, targeting the impairment has been a successful strategy for improving outcomes (10–17). In a head-to-head comparison of constraint-induced movement therapy (CIMT), which trains only the impaired hand, and bimanual training, the children in the bimanual training group had greater improvements in functional goal performance (18).

An exciting way to improve the engagement of rehabilitation is the use of robotics and gaming (19, 20). Robotic interventions can be tailored to a child's needs and goals, can record movement kinematics, and can provide movement assistance. Until recently (21), most interventions for children with unilateral CP using robotic type devices have been limited to unimanual training. Given the importance of bimanual training for this population, robotic or gaming devices that train bimanual activities could be of enormous benefit.

In this study, we piloted the use of the Bimanual Arm Trainer (BAT) in children with hemiparesis. The BAT was developed for stroke survivors. The BAT is comprised of two arms, with handles at each end for users to grasp. Training on the BAT involves making rowing motions to control a rowboat on a computer monitor centered between the arms. The two arms are coupled, such that a participant can drive movement of their more-impaired arm by moving their less-impaired arm. A pilot study showed that BAT training for 6 weeks in severely impaired adult stroke survivors led to improvement in Fugl-Meyer scores, and active range of motion in trained movements as well as distal untrained movements (22). Since the BAT provides bilateral movement training, we chose to test the feasibility of its use for children with hemiparesis. We believed that the combination of bimanual training capacity, ease of use, and engaging interfaces could be exciting and therapeutic for children.

We hypothesized that our BAT training protocol would be feasible and safe for children with hemiparesis. We also had two exploratory hypotheses: (1) BAT training would improve ROM; and (2) BAT training would improve upper limb movement in participants.

## 2 Methods

This study was approved by the Blythedale Children's Hospital Institutional Review Board (IRB) and the Biomedical Research Alliance of New York IRB. The study was listed on Clinicaltrials.gov (NCT03387449). Children were recruited between 2018 and 2020, then the study was halted by the global COVID-19 pandemic.

Children with hemiplegia were recruited from Blythedale Children's Hospital's day program and outpatient services. Children with hemiparesis (ages 5–17) were identified through electronic medical records. Caregivers of children with hemiparesis were contacted about the study. All caregivers provided informed written consent, and all children provided informed written assent.

Bimanual Arm Trainer device: The BAT was developed by Mirrored Motion Works (Cary, NC). It is registered as an FDA Class I exempt medical device: http://www.accessdata.fda.gov/scripts/cdrh/cfdocs/cfRL/rl.cfm?lid=461627&lpcd=PKS. The device facilitates movements of the affected hemiparetic arm by moving the unaffected arm at the shoulder and elbow joint. The device is interfaced with a video game for motivation and feedback. It also facilitates movements of the forearm and grasp and release on both the affected and unaffected sides.

*Study Design*: To test feasibility of the BAT, we employed a double baseline study design, such that all children would use the BAT. The intervention involved 18 study visits across 9 weeks, twice per week. We collected two sets of baseline measures: one was collected 9 weeks before the intervention began, then a second set of baseline measures were collected within a week before the beginning of the intervention. Children then completed the 9 week intervention, followed by post-intervention evaluations. Two children did not complete the first baseline assessment for logistical reasons.

*Inclusion criteria*: acquired Brain Injury at least 3 months prior to enrollment; unilateral hemiparesis; age 5–17 years; able to provide written assent, and a caregiver able to provide written informed consent.

*Exclusion criteria*: any medical issue that precluded compliance with the protocol; treatment with botulinum toxin or intrathecal baclofen in the 3 months preceding enrollment or plans to receive either during the intervention; implanted neuromodulatory or electronic device.

*Training Protocol*: The training protocol was designed to capitalize on principles of neuroplasticity and motor learning, including repetitive task-related movements and feedback [review (23)]. The training protocol, first developed for people with stroke (22), is rooted in the main postulates of the Bobath concept: (1) posture and movement are not separate entities, (2) sensory input influences motor output and (3) muscle strength does not necessarily equal function (24).

Participants completed two sessions per week, for nine weeks (18 sessions). If participants missed a session, every effort was made to reschedule the session. A typical session lasted 45 min, of which approximately 30 min were spent in active motion.

**Figure 1:**
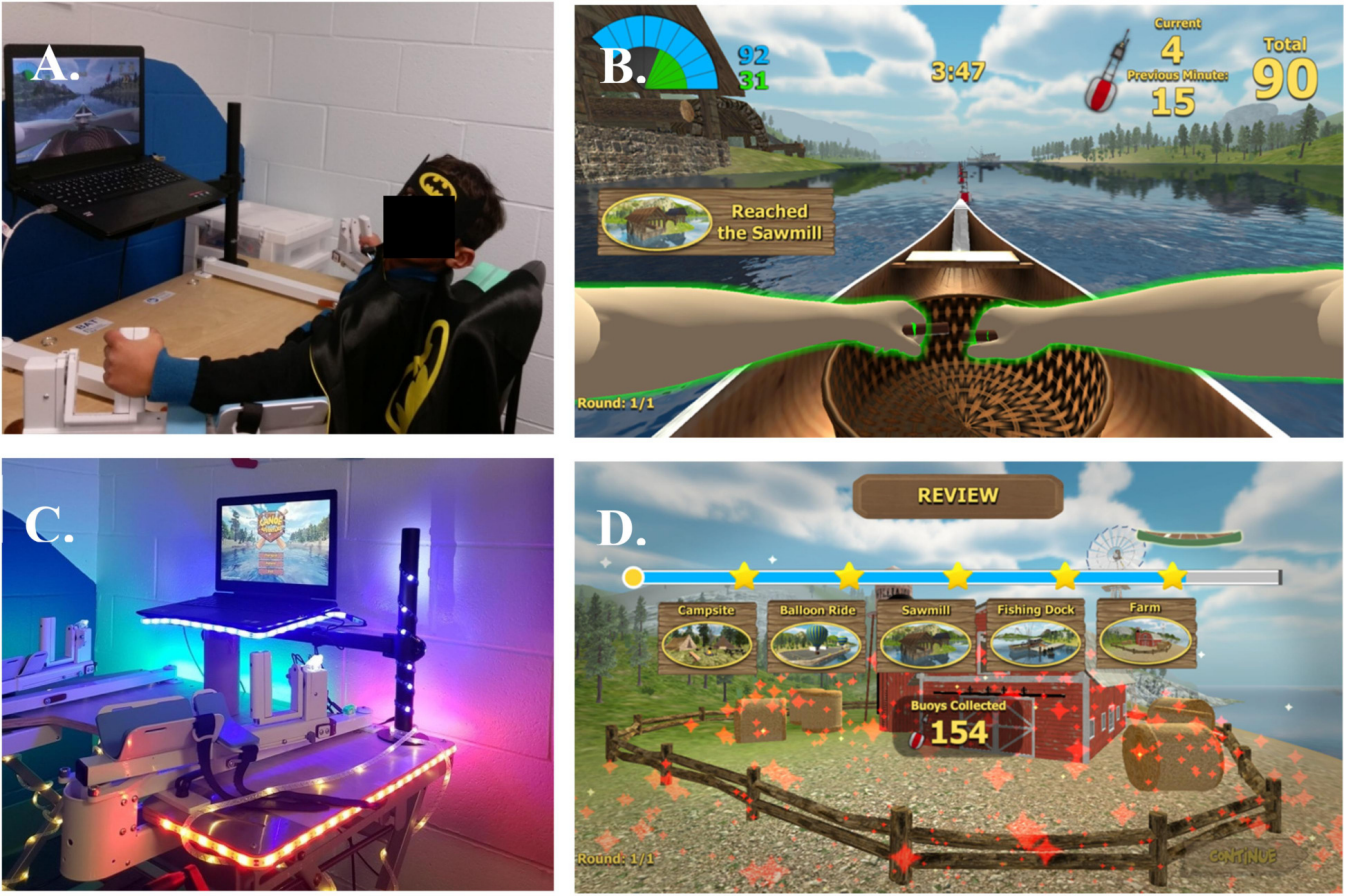
The BAT device. **(A)** A child demonstrates how to use the BAT. Users grasp the end of oar-like robot arms. **(B)** Gaming interface. Children played a rowing game, using the affected arm or both arms, by moving the robot arms in a rowing pattern. **(C)** Early in the study, a child informed US that the device was not a robot, because robots have lights. We added lights to the BAT. **(D)** The original gaming interface did not provide feedback about how far the user rowed along the river. Children wanted to know more. The device company added landmarks to the game, and added buoys that children would capture by passing the buoy. At the end of each session, children were provided a review of their river journey and a score of how many buoys they collected. This appeared to increase motivation and enjoyment of the intervention.

*Device-based bimanual-to-unimanual training* was provided with the BAT. The device provides bimanual-to-unimanual training of simultaneous shoulder external rotation and elbow extension, and independent training of pronation-supination and grasp and release of each hand. Range of motion and speed are recorded during training while feedback and motivation are provided through age-appropriate gaming modules (Figure 1).

The robotic device did not provide any movement assistance. During the session, children completed rowing using one of three conditions: rowing with only the affected arm, rowing with only the less-affected arm, and bimanual rowing.

*Clinical Assessments*: An assessor tested all subjects at the following time points: Baseline 1 (week 0), Baseline 2 (week 9), and Post (week 18). Videotaped assessments were scored by a trained, blinded evaluator. Some children had participated in other studies that included the same outcome measures as this study. If more than 9 weeks but less than six months had passed, we received consent and assent to use the final assessment of the prior study as Baseline 1 for this study.

### 2.1 Safety and feasibility outcome measures

*Safety*: Before and after each session, we asked if the child was experiencing pain or discomfort in their bodies, especially the arms and back.

*Subject Compliance*: The study coordinator monitored compliance with the training protocol, reviewed the training data, and optimized protocol adherence. Compliance was straightforward to enforce, as the training session was a workflow with several built-in checkpoints. At each checkpoint, the study coordinator was required to press a button to continue the session. A potential point of non-compliance would be if a child repositioned their arm on the robotic device. However, the children's arms were gently strapped into the robotic device using Velcro straps, minimizing the chance that the child's arm would become repositioned. Another point of non-compliance would be if a child did not cooperate during a session. All children completed all sessions. Brief breaks were given as needed.

*Feasibility*: We tracked the number of missed sessions and dropouts.

*Acceptability*: We asked children for feedback about the BAT design, gaming modules, and training.

### 2.2 Primary functional outcome measure

Assisting Hand Assessment (AHA): The AHA (25, 26) is an extensively validated test to measure bimanual hand use in children with unilateral upper limb impairments. The AHA measures the amount and quality of hand use during a play-like testing session. The AHA has excellent validity, reliability (0.97–0.99) and responsiveness to change (25). The AHA is sensitive to change in children with hemiparesis, with a minimal clinically important difference of 5 points (27, 28).

### 2.3 Secondary functional outcome measures

Box and Blocks Test (BBT): For the BBT, children sit at a table in front of a rectangular box divided into two halves. One side of the box contains 150 wooden 2.5 cm^3^ blocks (29). Children were asked to move blocks, one by one, using one hand, from one half of the box to the other, over a partition. The result is the number of blocks moved in 60 s. Each hand was tested. Inter-rater reliability is excellent (0.95). BBT is responsive to change in children with hemiparesis (30).

Jebsen-Taylor Test of Hand Function (JTTHF): The JTTHF measures the time taken to complete six unimanual tasks, which include flipping cards, moving small objects, and lifting cans. The total score is the amount of time taken to complete all tasks. The JTTHF is well-validated and has excellent reliability (30).

### 2.4 Robot-acquired outcome measure

Range of Motion (ROM): Active ROM was measured on the BAT before the intervention (baseline 2 timepoint), after the intervention (post), and each day of the intervention. ROM was not measured at baseline 1. The following metrics were generated by the BAT: arm rotation (shoulder/elbow), arm rotation angle, hand rotation, hand rotation angle, hand grip, and hand grip angle. The hand pieces of the BAT arms rotate freely.

*Data Analysis:* Data analysis was performed using GraphPad Prism software (GraphPad Software, Boston, MA). Due to the low n of the study, nonparametric statistics were used. The AHA, BBT, and JTTHF data were analyzed using the Friedman test, with *post-hoc* comparisons of timepoint differences. The ROM data were analyzed using the Wilcoxon Rank Sum test. Cohen's d, effect size, was calculated. *P* values greater than 0.05 were interpreted as statistically significant.

## 3 Results

Eight children with hemiparesis completed the intervention. The study was then halted by the COVID-19 pandemic. Table 1 contains demographic information for all participants.

**Table 1:** Participant demographics.

Participant ID	Age (y)	Sex	Race/Ethnicity	Affected upper limb	Diagnosis
BAT002	16	Male	Asian/Not Hispanic or Latino	Right	CP
BAT003	6	Male	Caucasian/Not Hispanic or Latino	Right	CP
BAT004	5	Male	Caucasian/Not Hispanic or Latino	Right	CP
BAT005	10	Male	Caucasian/Not Hispanic or Latino	Right	CP
BAT006	17	Male	Caucasian/Not Hispanic or Latino	Right	CP
BAT007	12	Female	Caucasian/Hispanic or Latino	Left	CP
BAT008	14	Male	Asian/Not Hispanic or Latino	Right	CP
BAT009	17	Male	Caucasian/Not Hispanic or Latino	Right	CP
BAT011	17	Female	Caucasian/Not Hispanic or Latino	Left	CP
BAT012	17	Male	Asian/Not Hispanic or Latino	Right	Acquired Brain Injury, age 16

### 3.1 Safety and feasibility outcome measures

As this is a feasibility study, we first assessed safety, compliance, feasibility, and acceptability of the BAT intervention.

*Safety*: There were no side effects or adverse events.

*Subject Compliance*: The study coordinator assisted the participants in completing the intervention and noted any deviations from the protocol. All participants completed all training sessions and were able to comply with the protocol.

*Feasibility*: There were no missed sessions or dropouts in the study. At times, we needed to reschedule study visits within a week to accommodate families' availability—for example, switching from a Monday/Wednesday schedule to a Monday/Thursday schedule. Children never did their two sessions per week on back-to-back days.

*Acceptability*: Children's feedback fit into three categories. (1) Physical appearance of the BAT; (2) arm movements required to complete the intervention; and (3) graphical aspects of the BAT software. Children of all ages gave feedback. Suggestions about the physical appearance of the BAT were implemented within a few days. The BAT software changes took a few weeks to implement.

1. The BAT platform has two components, rowing arms and a laptop, which are secured to a table (Figure 1A). One of the first children we enrolled did not agree that this was a robotic device, because, “Robots have lights.” We put strips of LED lights along the edges of the table and laptop, which the participants found appealing (Figure 1C). We also offered children a superhero cape and mask to wear during training, which children said increased engagement (Figure 1A).
2. Children had little trouble using the robotic arms. Some children suggested that the rowing should go faster, but increasing velocity would have possibly reduced the range of motion the child used to row the boat on the screen.
3. Children had many suggestions for improving the graphics of the rowing game. In the original BAT, made for adults with stroke, participants rowed a boat down a plain river and got little feedback on their progress during a session. Children were quite curious. “Where does the river go?” “Who lives by the river?” “Where are the fish?” “I feel like I’m rowing to nowhere!” The most common question was, “What's my score?”

The BAT programmers generously added many features to the interface. Landmarks were added to the shores of the river, such as a campfire and a sawmill. Every few rowing strokes, the children would collect a buoy from the river simply by reaching the buoy. No new movements were needed to collect the buoys. The children would see the number of buoys collected, which we called their score. At the end of each day, the children would see a summary of their progress (Figure 1D), showing the number of landmarks they passed and number of buoys they collected. Most children would ask us to record their scores, which became a goal to beat at their next visit. These modifications were appealing to children of all ages. However, some suggestions could not be implemented, such as making the BAT a two-player online game.

### 3.2 Primary functional outcome measure

**Figure 2:**
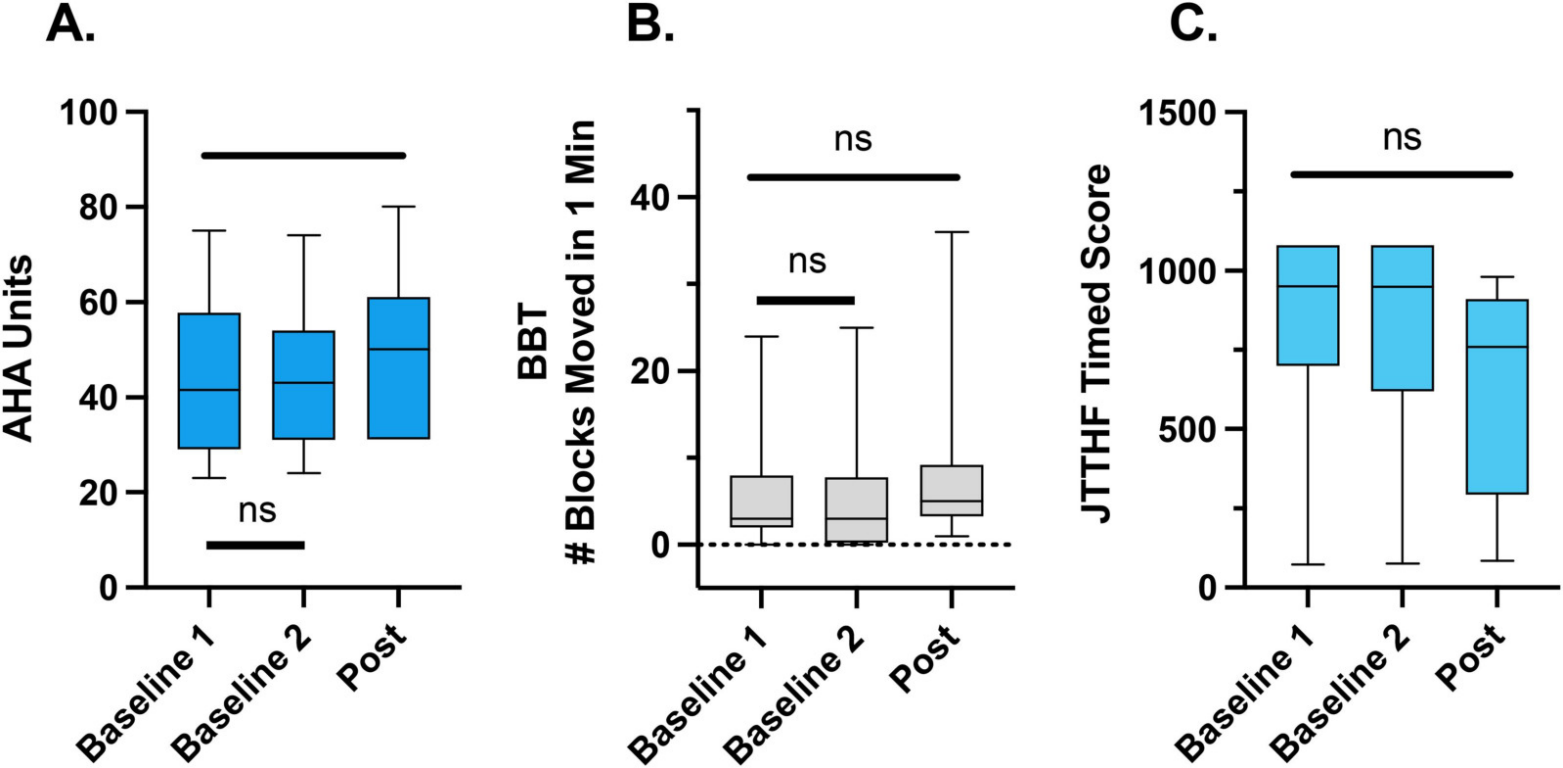
Clinical outcome measures (median, interquartile range, minimum, and maximum shown). There was a significant improvement in the AHA **(A)** from baseline 1 to post intervention. Tshere were no significant differences between the two baseline assessments. There were no significant changes in the BBT **(B)** or JTTHF across all time points **(C)**.

Our primary outcome measure assessed bimanual hand use. The AHA improved significantly after the intervention (Friedman statistic = 9.45, *p* = 0.0054, *d* = 0.32; Figure 2A). Baseline 1 and Baseline 2 were not statistically different (*p* > 0.99), while the post intervention scores were significantly improved from both Baseline 1 (*p* = 0.049) and Baseline 2 (*p* = 0.049). However, the change after intervention did not meet the minimal clinically important difference of the AHA, which is 5 points. This tempers the statistical findings.

### 3.3 Secondary functional outcome measures

Our secondary outcome measures assessed unimanual skill of the affected upper limb. The BBT, a measure of unimanual hand function, did not improve significantly after the intervention. Although the Friedman test showed an overall significant result Friedman statistic = 6.28, *p* = 0.049, *d* = 0.30; Figure 2B), *post-hoc* tests showed that none of the timepoints were significantly different from one another (Baseline 1 vs. Baseline 2, *p* > 0.99; Post vs Baseline 1 (*p* = 0.10); Post vs. Baseline 2 (*p* = 0.14).

Similarly, the JTTHF, a measure of unimanual hand capacity, did not significantly change after the intervention (Friedman statistic = 3.92, *p* = 0.16, d = 0.37; Figure 2C). The JTTHF is more challenging than the BBT, and some children could not complete the JTTHF in the time allotted (three minutes per subtest) before or after the intervention.

Range of Motion (ROM): The robot delivered pre- and post-intervention evaluations of range of motion for the arm and hand. Here we report ROM of the affected arm and hand. Arm rotation did not change after intervention (Pre 52.5 ± 8.7, Post 50.4 ± 15.1, *p* = 0.063). Similarly, arm rotation angle did not change after intervention (Pre 10.2 ± 2.4, Post 9.2 ± 3.1, *p* = 0.063). Hand rotation (Pre 3.8 ± 2.7, Post 3.8 ± 3.2, *p* = 0.13) and the angle of hand rotation (Pre 0.5 ± 0.58, Post 0.5 ± 0.58, *p* = 0.50) also did not change significantly after the intervention. Lastly, neither hand grip (Pre 4.2 ± 2.4, Post 4.2 ± 1.9, *p* = 0.13) nor the angle of hand grip (Pre 4.1 ± 2.1, Post 0.5 ± 0.6, *p* = 0.25) changed significantly after the intervention.

## 4 Discussion

Children with hemiparesis completed an intervention using a novel robotic device that engages both upper limbs in repetitive movements. The goal of this study was to determine feasibility of the BAT for children with hemiparesis. Two children were enrolled before we added the nine-week control period (B1) preceding the nine-week training period. Aside from these two children missing the B1 visit, all children completed the protocol and assessments. We had hoped to test feasibility in a larger cohort, but we needed to stop the study for the COVID-19 pandemic and were unable to restart the study after the pandemic.

The primary outcome measure, the AHA, did not change during the control period, and improved significantly after 18 sessions of training. While the primary outcome measure met statistical significance, it did not change more than the minimal clinically important difference of 5 points. Therefore, this pilot study shows promise for improving bimanual hand use, but may need either a larger number of sessions or more densely scheduled sessions to boost improvements beyond 5 points.

The secondary outcome measures of unimanual skill showed mixed results. The BBT showed an overall statistical change, but none of the time points were significantly different from one another. The JTTHF did not significantly change across the control or training parts of the study. The JTTHF is more difficult than the BBT to perform, and four children could not complete the JTTHF in the allotted time of 1,080 s. There is insufficient evidence to draw conclusions about the impact of BAT training on unimanual skill. A larger cohort of participants is required.

Range of motion did not significantly change after the intervention. This is in contrast to the pilot study using the BAT as an intervention for severely impaired stroke survivors (22). In that study, improvements in ROM were found after the intervention. The children in this study were more mildly impaired than the adults in the stroke study. It is possible that the BAT could improve ROM in children with more severe impairments. It is also possible that children require a more intensive intervention to result in ROM improvements. This is discussed below, with other recommendations for future studies.

Although our sample size is too small to perform subgroup analyses, it is of note that the three most impaired children showed improvements in the AHA that surpassed the MCID. On the BBT, two of these children could not move a single block from one box to the other in one minute, and the third individual moved one block. This represents severe impairment. It is promising that these three children were able to use the BAT and showed clinically meaningful improvement in bimanual use.

Gamification of rehabilitation can improve adherence and engagement of children (19, 20). However, we found that enjoyment involves more than gamification. We encouraged our participants to share ideas and preferences with us. We are grateful that the creators of the BAT were able to update the gaming environment based on feedback from the children. For example, children were frustrated that they did not know where their rowboat was going, they did not know who lived along the river, and they did not get a score. The BAT creators added labeled landmarks to the riverbanks, and assigned scores related to how long and far a child was rowing.

We also made changes to the exterior of the BAT. After a child insisted that robots must have lights, we affixed a row of small lights to the frame of the BAT, as shown in Figure 1. Children enjoyed giving feedback and were pleased when we made their suggested changes. We urge other researchers to ask children their opinions about interventions in which they enroll, and to implement changes when possible. Children and their caregivers deserve the opportunity to share their suggestions.

With the emergence of new technologies for neurorehabilitation, it is important to target interventions to specific impairments common to the population being studied. The BAT encourages repetitive symmetrical movement of the arms. As such, the movement of a child's less-impaired arm encourages and assists movement of the more-impaired arm. This enables children with severe impairments the ability to move their more-impaired arm, as it is yoked to the other arm during part of the training.

Training intensity and repetition are key factors in rehabilitation (1, 11, 18, 24, 31–33). It can be difficult to provide children optimal intensity and repetition in clinics or schools. Bringing rehabilitation technology into the home provides the opportunity for more practice. The BAT is small, portable, and has potential to be used in the home.

There were several limitations to this study:

We had hoped to test feasibility in a larger cohort, but we needed to stop the study for the COVID-19 pandemic and were unable to restart the study after the pandemic. This left us with a low number of participants, of a wide range of ages, which decreases the impact of our findings.

We did not include a control group, rather, we included a double baseline, so that all participants would use the BAT. A next step in testing the efficacy of the BAT would be to conduct a properly powered randomized controlled trial (RCT). We also did not include a follow-up assessment. It is important to know whether the gains children made after the intervention are long-lasting. An ideal RCT would include a follow-up evaluation. Another limitation is that we did not have an unblinded assessor, although the AHA was scored by a blinded evaluator. This was due to staffing and budget issues. Future studies should engage blinded assessors in all aspects of the intervention. There are many promising future directions. Optimal dosing and delivery schedules need to be developed, which may differ based on impairment level of participants. Since the BAT is portable, testing efficacy of home-based use could provide children and their families a more convenient method of training. In future studies, two different training groups could be compared, such as different dosing, different delivery schedules, or home- versus lab-based training. Studying the correlates of training on brain plasticity would be important for optimizing the training parameters—using non-invasive brain mapping or functional magnetic resonance imaging could inform the understanding of neural correlates of training.

## 5 Conclusion

This study showed that the BAT intervention is feasible for children with hemiparesis. Children showed improvements in bimanual hand use, though not to the level of the clinically meaningful difference. Further work is needed to determine the optimal treatment schedule and ideal participants for rehabilitation with the BAT.

## Data Availability

The raw data supporting the conclusions of this article will be made available by the authors, without undue reservation.
